# Treatment seeking behavior of people with malaria and households’ expenditure incurred to it in a block in endemic area in Assam, North East India

**DOI:** 10.1186/1471-2334-12-S1-P41

**Published:** 2012-05-04

**Authors:** Dhruba Jyoti Borah, P Sankara Sarma

**Affiliations:** 1 Regional Coordinator for North Eastern States (Care Support and Treatment), NACO, India; 2 Department of Statistics, AMCHSS, Sree Chitra Tirunal Institute for Medical Science and Technology, Kerala, India

## Background

The 60-90% preponderance of *Plasmodium falciparum* malaria places an economic burden in the community. The objective of the study was to know the treatment seeking behavior of people with malaria and households’ expenditure incurred to it in a block in endemic area.

## Methods

In a cross sectional survey, all 210 diagnosed malaria patients within one month were interviewed through a pre-structured interview schedule during the high transmission season in pre, post and monsoon period in 2010.

## Results

During the last episodes of malaria, 58.5% sought treatment from government health facilities out of which 41.4% went to allopath, 17.1% went to community health workers, 25.3% went to private practitioners of which 12.9% went to tea garden doctors, 8% to other facilities, 7.6% to traditional healers, 9% to homeopath and .4% to none. Self treatments were taken by 59% patients. *Plasmodium falciparum* affected 55.2% patients, Vivax 41.4% and mixed infection 3.3%. The median expenditure incurred on treatment was `821, on preventive action were `150 and daily wage loss was `100. The SC, ST (65.7%), farmers and daily wage laborers were going less to government facilities. The household expenditure was mainly associated with self treatment and repeat malaria.

## Conclusion

The people of lower socio-economic group utilized more government health facilities for malaria treatment. Self treatment and repeated malaria had an impact on household expenditure. Improvement in quality health care delivery in public sector and IEC activities in the community would empower for maximum utilization of government health facilities.

